# Questions for more insightful and useful analyses of barriers and facilitators to public involvement and engagement in health research: a critical narrative review

**DOI:** 10.1186/s40900-026-00963-9

**Published:** 2026-09-01

**Authors:** Lauge Neimann Skov

**Affiliations:** 1 Department of Public Health, University of Copenhagen, Øster Farimagsgade 5, Copenhagen, 1353 Denmark; 2 Department of Education, Steno Diabetes Center Copenhagen, Borgmester Ib Juuls Vej 83, Herlev, 2730 Denmark

**Keywords:** Patient and public involvement, Participatory research, Health research, Barriers, Facilitators, Challenges, Methods, Methodology, Research questions.

## Abstract

**Background:**

Public involvement and engagement in health research is widely promoted yet remains challenging and unevenly practised across health research contexts. To explain this, it has become popular to analyse so-called barriers to and facilitators of involvement. However, critics argue that such analyses are simplistic and generate little knowledge to guide action. This review critically appraises the typical form of barrier analysis concerned with public involvement in health research and proposes eight lines of inquiry that may make future analyses more insightful and useful.

**Methods:**

This is a critical narrative review informed by a systematic search of four health research databases in June 2026. Records were eligible if they stated a research objective concerning barriers to public involvement in health research and reported their findings in a topical manner, meaning that they mainly named and summarised findings and organised them in themes. These topical barrier analyses were critically appraised in relation to their capacity to improve understanding and inform involvement practice, and their findings were contrasted with broader debates about public involvement.

**Findings:**

Eighty topical barrier analyses concerning public involvement in health research were identified and reviewed. The review argues that the analyses pay little attention to what a barrier is or means, what it obstructs, when and where it arises, and whom it affects. Their recommendations are abstract, basic or largely aspirational; they link irregularly to the barriers identified; and they are usually directed towards researchers while doing little to help members of the public. The review argues that these shortcomings stem from a focus on two repetitive questions: what are the barriers, and how might they be overcome? To move beyond the analytical shortcomings, the review proposes eight new lines of inquiry: five aimed at making analyses more insightful and three at making them more useful.

**Conclusion:**

Unlike critics who call for barrier analyses to be abandoned, or those who call for methodological refinements, this review argues that the future of barrier analyses depends on a willingness to ask new questions that may generate knowledge that is more insightful and useful than what is already known.

**Supplementary Information:**

The online version contains supplementary material available at https://doi.org/10.1186/s40900-026-00963-9.

## Abstract (significance)

In health research, people are often enthusiastic about the public playing a more active role rather than being treated as test subjects. In practice, however, both members of the public and researchers find it challenging to collaborate. A common way to understand and deal with the difficulties is by making studies that identify so-called barriers and facilitators. Barriers are things said to obstruct involvement, while facilitators are things said to help it happen. In this paper, I critically discuss what such studies show and whether they offer useful guidance.The paper focuses on eighty studies that, in a simple manner, summarise what people have said about barriers and sort these findings into broad topics like "lack of time" or "lack of funding". I find that these studies repeat familiar findings across very different health research projects. Although such findings may be true, they do not tell us much about what a barrier is, what forms of involvement it blocks, where and when barriers appear, or for whom. The recommendations these studies give are often vague or basic, fail to address identified barriers, and support researchers more than members of the public.This happens because the studies keep asking the same questions: what are the barriers, and how can they be overcome? To avoid future studies confirming what we already know, I propose questions that may make them more insightful and useful for those who seek to promote public involvement in health research.

**Supplementary Information:**

The online version contains supplementary material available at https://doi.org/10.1186/s40900-026-00963-9.

## Introduction

The challenges of involving members of the public in health research are many, and they have been given many names: dilemmas, mess, and costs, to name a few. More commonly, however, the challenges are talked about as barriers. This paper revolves around these so-called barriers and, more specifically, the limits to the typical way we try to get a grip on them: by asking what the range of barriers is and how they might be overcome to advance public involvement in health research.

In this paper, I use the phrase *public involvement and engagement in health research* or similar shorthand to refer to research where people with experience of, or interest in, the research topic participate in and contribute to all or some aspects of the process – or, put more simply, to refer to research carried out *with* or *by* these people rather than to, about, or for them [1]. Such participatory approaches position members of the public as consultants, collaborators, co-researchers, or even directors of health research, rather than merely as test subjects, respondents, or informants.

In general, people speak about public involvement as something self-evidently good [2, 3]. Research projects that actively involve members of the public are said to become more democratic, just and innovative, while the resulting interventions are said to become more effective and relevant [4]. Back in 2000, scholars proclaimed a “growing enthusiasm” for consulting members of the public across all stages of research projects [5]. Twenty years later, the “enthusiasm for involvement [had] never been higher” [6] and was declared both “widely accepted” [7] and “increasingly popular” [8]. Today, public involvement continues to be called “an increasing priority in health research” [9] and, indeed, there are now notable health organisations which promote involvement and engagement [6, 10], including funders that require researchers to declare their intentions or plans to engage the public [11, 12], and academic journals that insist that authors state whether and how they involved members of the public in the research process [13, 14]. In this situation, few people dare to argue – at least in public – that there are situations in which the public’s role should be limited (for the rare exception, see 15).

Despite the outspoken support, public involvement is far from a universal standard in health research. In BMJ-affiliated journals, the proportion of papers reporting some kind of public involvement has risen from 0.5% of all papers published in 2013-14 [16] to 22.6% of the papers published in 2020 [17]. For systematic reviews, the number was as high as 44.4% in 2022 [18]. Such trends may be encouraging, but there is other evidence that is less so. One systematic review found 23 trials published between 2011 and 2016 with public involvement and engagement and compared this number with the 371,159 trials registered in the Cochrane Central Register of Controlled Trials during the same period [19]. For some research fields, systematic reviews find little to no evidence that members of the public have been involved in research at all [20, 21]. So, while public involvement and engagement may be spreading, it does not happen evenly across research fields, nor does it happen as quickly as its proponents would like.

It is as if something is obstructing participatory progress, albeit in some contexts more than in others. Indeed, many know that public involvement can be and often is challenging, for moments or for periods, in one way or another, for some more than others, and to varying degrees. These experiences resonate throughout the academic literature in which they are presented as dilemmas [22], tensions [23], paradoxes [24], complexities [25], costs [26], mess [27], muddle [28], perplexity [3], threats [29], or social pollution [30]. But most of all, the challenges of public involvement and engagement are framed as *barriers* to be overcome. Barriers can include a lack of time and funding for involvement, researchers’ scientific language and jargon, a lack of knowledge among members of the public about research processes and methods, and concerns about the bias and representativeness of those involved [31]. To counter such issues, researchers are advised to be culturally sensitive, explain their research methods, appoint someone to facilitate the process, and secure adequate funding for involvement. Findings and recommendations of this kind are the results of what I will refer to as *barrier analyses*.

### Barrier analyses: what should we do with them?

*Barrier analyses* rely on a simple – some say simplistic [32, 33] – framework used to identify barriers that are negative for public involvement in health research and facilitators that are positive. This framework can be applied to qualitative [34], quantitative [35] or mixed-methods data [36] with the aim of fostering insights that are then meant to be translated into recommendations for policy and practice. To this end, barrier analyses have become so popular that they are now a “default” [34] analytical approach in health research and beyond [37, 38].

Following the popularity of barrier analyses, some scholars have pointed out that these analyses tend to suffer from substantial shortcomings [32–43]. The consensus is that they are products of a largely common-sense analytical approach that tends to reduce complex realities to lists of “good things” and “bad things” [34], privileging “the salient, uncontroversial, and easily communicated factors… [over] those that are complex or unanticipated” [36]. Another criticism is that barrier analyses do not deliver on their practical promises but offer “very limited advice about how to intervene in practice to secure better outcomes” [41]. As a result, the most radical critics deem the approach inherently flawed and call for barrier analyses to be completely “abandoned” [43], “discarded” [41] and “retired” [40]. 

More constructive critics argue that barrier analyses can – and must – be conceptually and methodologically refined to remain relevant [34–38]. A prominent recommendation is that barrier analyses need to define what they mean by barriers and facilitators, which many currently fail to do [35–37, 42]. Other recommendations call for more rigorous research methods and transparent reporting of the results [35–38], and for the development of new theories or more consistent use of existing ones [34, 35, 37, 38]. More participatory approaches have also been proposed as a way to improve barrier analyses [34], which advocates for public involvement will find particularly relevant.

All these potential improvements are worth considering, but they also risk undermining the strength of barrier analyses: their simplicity. Barrier analyses appear to be relatively straightforward to undertake and their results relatively easy to grasp, even for people without formal research training. The future of barrier analyses thus involves a tension between academic rigour and public accessibility. This tension will be particularly acute in the context of public involvement, where greater conceptual and methodological sophistication might exclude the very members of the public whose participation these analyses are intended to promote.

### Redirect barrier analyses rather than abandon them

The debates over the concepts and methods, however, are largely concerned with the *means* of barrier analyses. Equally important, yet less frequently discussed, is whether barrier analyses are guided by the right questions in the first place. 

Today, barrier analyses are occupied with the same twofold questions: What are the barriers to a given intervention or policy? And how can they be overcome? This fixation has generated “wideranging” [40]and “seemingly endless” [37] lists of barriers and facilitators, as study after study identifies familiar factors, although in unique configurations [37]. As Haynes and Loblay argue, these “are far from the *only* questions we should be asking” [34].

In this review, I propose eight lines of inquiry regarding public involvement in health research that are neglected by barrier analyses yet crucial for anyone who seeks to increase and diversify the ways that members of the public contribute to health research. The review’s ambition is to inspire a more insightful and useful barrier literature. By *more insightful*, I mean a literature more capable of telling us something genuinely new about barriers to public involvement compared to what is already known. By *more useful*, I mean a literature more likely to guide us in how to advance a participatory vision for health research. This ambition cannot be achieved by restating or reorganising what is reported in the existing barrier literature as is the approach in aggregative reviews [44]. Instead, I offer a review of barrier analyses which interprets and critiques the literature in order to expose underlying assumptions and identify overlooked questions, so that we may move beyond the findings it explicitly reports. This makes for a two-part paper: a review, in that it is based on a body of literature identified through a systematic literature search, and arguments about that literature, which interprets and move beyond the literature itself. What follows is therefore not an inventory of the barriers to public involvement reported to date. Readers looking for such lists can find a great many such lists among the studies reviewed here. To be clear, the main goal is neither to abandon barrier analyses or to refine them methodologically and conceptually. The goal is to redirect barrier analyses and drive them towards lines of inquiry that are less concerned with the range of potential barriers and more concerned with their meanings, contexts, and functions.

Before outlining the methodology of the review, it is worth noting that this review was conducted without formal public involvement. Patient partners were invited at the outset but declined, partly because available funding was insufficient to compensate them adequately and partly because they preferred to engage in projects focused directly on their health condition rather than on involvement as a topic. I could frame these issues as barriers, but, as the review will propose, there is more to it than that.

## A critical narrative review approach

This paper is a critical narrative review. Narrative reviews may be thought of as “a scholarly summary along with interpretation and critique” [45], and they place particular emphasis on careful reading and reflective writing [45, 46]. It is critical because it appraises the barrier literature in terms of what it does not ask and what it takes for granted, rather than how well it addresses the questions it already poses [44, 47]. The appraisal thus provides a basis for moving barrier analyses concerning public involvement in health research beyond their current analytical shortcomings.

The review has been shaped by a prolonged and iterative (i.e. hermeneutic [48] engagement with a broad selection of studies that analyse barriers and facilitators to public involvement and engagement in health research. It adopts an interpretivist approach [44, 47, 48], meaning it is informed by my interpretations of the literature. At the same time, I sought to develop arguments that would resonate more widely with people undertaking or studying public involvement in health research, especially those who are already familiar with barrier analyses or preparing to conduct such analyses themselves.

### Scope of the review

This review covers a substantial but selective body of literature on barriers to public involvement in health research. It addresses analyses which explicitly (1) state a research objective or question about barriers to public involvement in health research and subsequently (2) report their findings in a topical manner. When using the term *topical*, I – somewhat inspired by other scholars[49, 50] – mean a type of descriptive analysis that presents barriers and facilitators to involvement based on qualitative data, existing literature, or the authors’ own experiences with public involvement. Topical findings are reported in a list-like format with each barrier and facilitator presented separately, one after another. The level of detail varies, ranging from brief descriptions in a single paragraph that merely name the barriers and facilitators within papers addressing multiple research questions, to extensive accounts in which barrier and facilitator findings constitute the sole focus of the paper. I chose to focus on the topical format because it appears to be the dominant approach to reporting barriers and facilitators to public involvement. By focusing on this format, the review excludes other types of barrier analyses, for example, those in which barriers and facilitators have been measured and are reported based on quantitative data or those that describe barrier encounters in a more narrative manner. I return to this limitation in the discussion.

To collect relevant topical barrier analyses, I first searched for relevant records in four health databases (MEDLINE, Embase, CINAHL, and PsychInfo) in June 2023. Knowing that even validated and complex search strings struggle to identify all relevant literature on public involvement and engagement [51], I developed relatively simple search strings using Boolean operators to combine relevant keywords for *barriers*, *involvement* or *engagement*, and *health research*. The search strings targeted truncated terms for involvement and engagement to identify barrier analyses regarding a wide range of involvement and engagement approaches (see the supplementary material). The search was updated in June 2026.

In total, the search identified 2,269 unique records. These were first screened by title and abstract and, when seemingly relevant, as full-text papers. I excluded posters, protocols and abstracts and considered papers eligible regardless of their language of publication (a flow diagram is available in the supplementary material). For papers published in languages other than English, I translated the texts into English using DeepL Translate [52]. All records were screened in EPPI-Reviewer Web [53] and data to describe the key characteristics (e.g. publication year, journal, research design, and public involvement terminology) of the literature were extracted into Microsoft Excel.

### Critically appraising the literature

The included papers were read repeatedly to develop a critical appraisal of barrier analyses regarding public involvement in health research. The appraisal was informed by a broader selection of literature on public involvement. This literature was not identified through a formal literature search. Instead, this literature reflects my knowledge gained from following the field since 2017 and my judgements of what is analytically (and rhetorically) relevant. To select literature in this manner would be inappropriate for, say, a systematic review but is accepted – even encouraged – within interpretivist review traditions that emphasise interpretation and imagination over procedural rigour [44, 47, 48]. This is also why the paper can also be read as a critical essay grounded in a body of literature more than as an attempt to summarise that literature. Wherever the appraisal draws on broader literature, I explicitly cite the sources. Readers may be inspired – or provoked – to consider other sources of knowledge they regard as more pertinent. I would welcome any such reactions since the aim is not to draw definitive conclusions but to encourage reflections on what questions would make barrier analyses more insightful and useful than they currently are.

## Characteristics of the topical barrier analyses reviewed

**Fig. 1:**
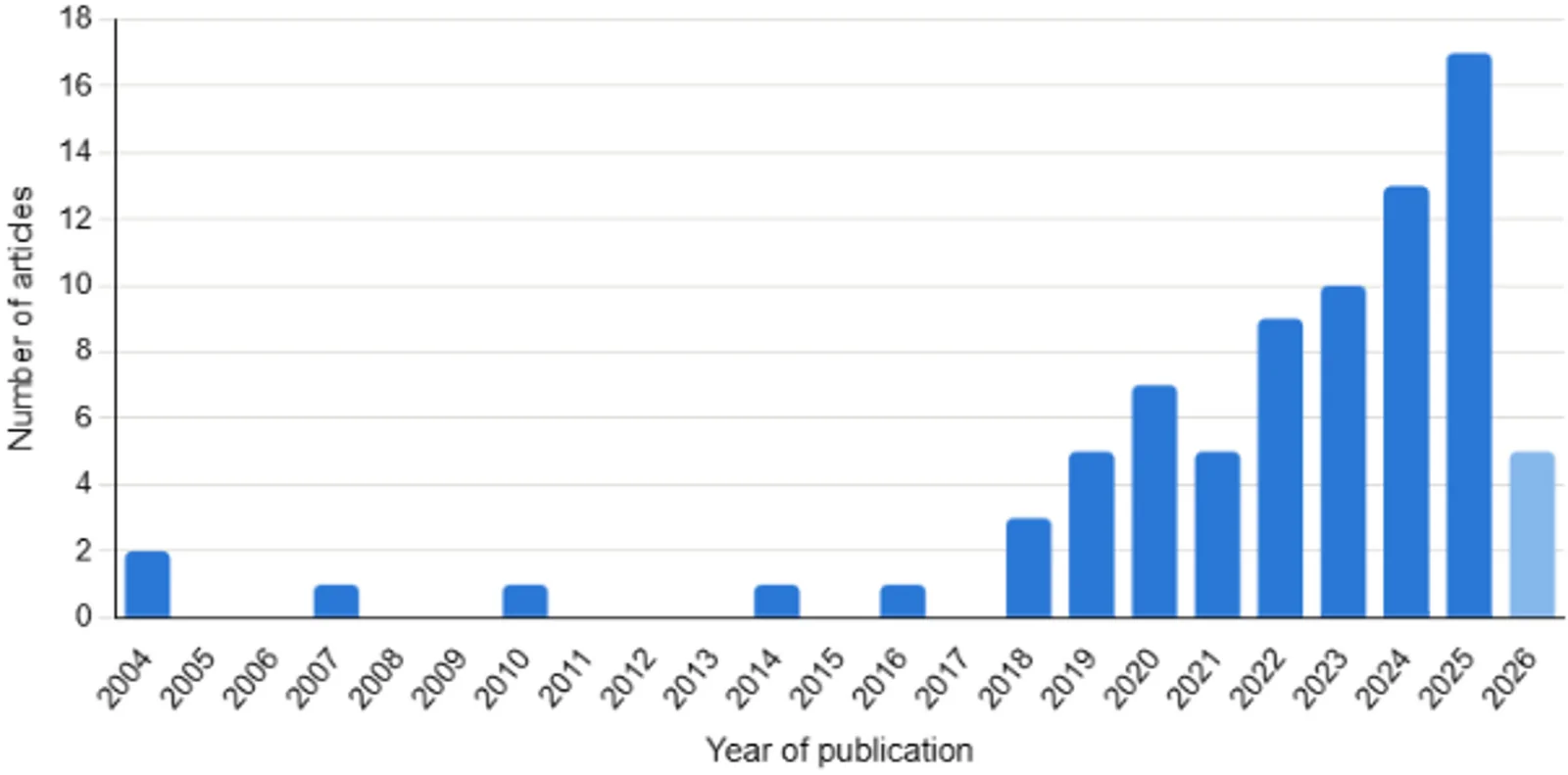
Number of barrier analyses by year of publication. The last bar in lighter blue represents only the first half of 2026 because the literature search was done in June 2026

In total, eighty topical barrier analyses regarding public involvement in health research were reviewed [54–133]. The first were published in 2004 [77, 89], with the publication rate having risen markedly since 2018 (Fig. 1). Over time, the barrier analyses have appeared across journals representing a range of health research fields. Around half of the identified barrier analyses are the only such analysis for their respective journal, but journals dedicated to public involvement have published several topical barrier analyses, with *Research Involvement and Engagement* publishing the most [56, 57, 59, 80, 85, 87, 107, 108, 110, 122–124, 131, 132], followed by *Health Expectations* [65, 72, 81, 95, 101, 102, 106].

A wide range of research designs have been used to generate the topical barrier analyses. Forty-two report primary research with original data [54, 66, 67, 69, 74, 77, 78, 80–88, 92–97, 99–104, 107–110, 114, 116, 119, 120, 123, 125, 127, 128, 130–132], most frequently collected through interviews [54, 66, 67, 69, 74, 77, 78, 81–85, 87, 88, 94–97, 102–104, 109, 114, 116, 123, 125, 127, 128, 132] and focus groups or workshops [67, 74, 86, 93, 94, 99–101, 107–110, 120, 123, 130, 131]. Twenty-eight report secondary research, i.e. they are literature reviews (55, 57, 62–65, 68, 70, 72, 73, 75, 76, 79, 89–91, 98, 101, 106, 115, 117, 118, 121, 122, 124, 129, 131, 133], with most labelled as scoping [63–65, 72, 76, 98, 115, 118, 121], systematic [55, 57, 73, 79, 106, 122, 124, 133], or narrative [31, 75, 91, 131] reviews. Finally, ten topical barrier analyses are based on researchers’ personal reflections on or experiences of barriers to public involvement and engagement in health research [56, 58–61, 105, 111–113, 126].

The barrier analyses vary in terms of their health research context and public involvement terminology. Fifty-two address involvement and engagement in the context of health or mental health research broadly speaking, with the remaining twenty-eight situating barriers to involvement within research on specific health-related conditions such as dementia [70, 118], autism [125], leprosy [66], cancer [81, 88, 97, 112], child maltreatment [91, 115], obstructive sleep apnoea [102], and substance use [61, 93]. The analyses represent diverse approaches to or terms for public involvement, although the vast majority adopt terms that refer to a more or less specific social group being involved, engaged or both (e.g. patient engagement [55, 58, 63, 66, 76, 87, 95, 107, 116, 122, 126, 132], youth involvement [96, 101], or involvement of people who use drugs [61]. Seven analyses use partnership-oriented terms (e.g. patient partnership [56, 72, 105] or academic-community-government partnerships [88]; eight specifically use the term community-engaged research [69, 93, 98, 109, 117, 119, 128, 130]; while fifteen use other distinctive terms such as citizen science [94], co-design [64, 103, 106], participatory research [115, 125], and lived experience research [60]. Finally, members of the public authored or co-authored thirty-eight of the topical barrier analyses reviewed [56–61, 63, 66, 70, 72, 74, 75, 77, 78, 80, 82, 83, 87, 89, 93, 96, 101, 102, 104, 105, 107, 108, 110–113, 116, 122, 126, 130–133].

Table 1 illustrates some typical findings, slightly edited for brevity, reported in the three most frequently cited topical barrier analyses reviewed.

**Table 1:** Barriers and facilitators to public involvement and engagement in health research from the most cited barrier analyses

# of citations^a^	Study	Barriers	Facilitators^b^
1,085	Domecq et al. [55]	**Logistics**: time needed to complete the research; time constraints of patients and researchers; and funding for patient engagement. **A worry about tokenism**. **Scope creep**: a concern that engagement may include irrelevant community concerns and make the research unfeasible.	**Adequate time** to build reciprocal relationships. **Mutual respect**. **Clear expectations** documented in study protocols.
194	Oliver et al. [77]	**Poor representation**: consumers are a minority and feel it is unclear whether they should express their own views or those of a consumer constituency. **Unfamiliarity** including consumers’ unfamiliarity with agenda-setting tasks and procedures or with the people they work with, as well as with overly technical discussions; and researchers’ unfamiliarity with consumer-centred approaches. **Working relationships**: consumers feel isolated and feel like outsiders. **Attitudes**: consumers think professionals regard professionals’ own views as more valuable; staff report that consumers are resistant and expect professionals to be the experts; and consumers are cynical and critical. **Time constraints**: time is insufficient to reach common ground; deadlines between meetings are not agreed in advance; a lack of notice before events. **Communication**: information is insufficient or too general; consumers are disappointed about a lack of feedback; staff do not have time to brief individuals; the use of jargon and acronyms.	**Leadership**: programme directors and committee chairs commit to consumer involvement, and consumers chair workshops. **Breadth of involvement**: recruit consumers via questionnaires and market researchers, use networks, travel around the country, and combine a range of methods to involve consumers. **Working relationships**: build on established relationships, face-to-face meetings, and interactions before and after workshops, including social aspects such as lunchtime, and staff encouraging consumers, and consumers supporting each other. **Communication**: send event information in advance and follow-up information later; make face-to-face meetings and telephone calls rather than send letters and emails alone; use plain English language; avoid technical jargon; and use languages other than English, when needed. **Training and support** that complement consumers’ experiences. **Programme resources** for involving consumers, including policies for paying honoraria and time to think and discuss. **Build on experience** from ongoing working relationships to develop consumers’ skills and confidence and increase clarity around consumer involvement through procedures, guidelines, and job descriptions.
157	Boote et al. [31]	Tensions between stakeholders with **conflicting agendas**. **Public understanding of health research methods** including concerns about whether members of the public understand and accept trial methodology, especially randomisation. **Time and cost**: difficulty obtaining funding for involvement at the early research design stage. **Representativeness**: members of the public are not a random selection but highly interested in the research topic and have characteristics different from potential research participants. **Language and jargon** required by funders and commissioners may put off members of the public.	**Cultural sensitivity** including interviews conducted by local people using question guides translated into the local language. **Clear explanation of health research methods**, including oral presentations and education of communities in the proposed research methods. **Independent facilitation**, e.g. by employees at health charities or generalists, rather than specialist investigators. **Funding for involvement at the research design stage** to cover expenses and payment for members of the public.

^a^ Number of citations, according to Web of Science, on 5 May 2026

^b^ Oliver et al. and Boote et al. used the term “facilitators”, whereas Domecq et al. used the term “solutions”

The characteristics of topical barrier analyses concerning public involvement in health research illustrate their proliferation. However, the purpose of this review is not simply to describe and summarise the literature. Rather, it is to critically evaluate its analytical limitations in order to identify new directions for inquiry as I do in the next section.

## Eight shortcomings of the topical barrier literature

So far, I have described how I collected a body of barrier literature to review: specifically, topical analyses that enumerate barriers and facilitators to a diverse range of public involvement approaches or terms. I have shown that the topical format has been used in combination with various research designs across a range of health research contexts. Having reviewed 80 topical barrier analyses, I – like others [33, 34, 37, 38] – find that they have become repetitive, leaving several questions unaddressed.

In what follows, I call topical barrier analyses into question in ways that create opportunities to pursue new lines of inquiry. As noted earlier, I relate the barrier analyses to, and contrast them with, insights and debates in the broader literature on public involvement and engagement. In doing so, I refer to the topical barrier literature, not always exhaustively but sufficiently to illustrate and support my arguments.

The appraisal is organised into eight subsections. The first five mostly evaluate how insightful barrier analyses are, while the last three evaluate their practical value. In brief, I argue that future barrier analyses should begin to examine more closely what *a* barrier is and can mean; what, precisely, it obstructs; and where, when, and for whom it is present. Additionally, I point out that barrier analyses can be more specific about what facilitators and recommendations are meant to do analytically and practically. Finally, I find reason to begin questioning how barrier analyses can become useful to members of the public and not only researchers. The appraisal forms the basis of the subsequent discussion in which I more clearly propose questions and point out a few non-topical barrier analyses which all look beyond the repetitive pattern of topical barrier analyses. 

### What is *a* barrier?

Topical barrier analyses commonly state that they aim to identify barriers to public involvement in health research. To identify barriers to public involvement, one must have an idea of what *a* barrier is.

Yet only five topical barrier analyses provide anything resembling a definition of the concept on which each analysis rests. Three topical analyses briefly describe barriers as factors that “disadvantaged” [77], “challenged or prevented” [86] public involvement, or factors which “interviewees identified as impeding” [128] involvement. Another analysis refrains from providing an explicit definition and simply states that it used the barrier construct from the Health Belief Model, citing a source [119]. The fifth attempts to distinguish barriers from constraints, defining the former as issues that can*not* be overcome and the latter as issues that can [76]. However, this distinction quickly collapses when the findings are presented and the authors concede that some factors may function either as barriers or as constraints “[d]epending on the study context” [76] – without specifying what these contexts are.

In the absence of clear definitions, topical barrier analyses frequently use terms like challenges, tensions, limitations, hurdles, and concerns as synonyms for “barriers”. This also happens in some papers’ sub-titles, and it can sometimes be difficult for a reader to identify where the barrier findings are reported. Other analyses even report separate findings on both barriers and challenges [63, 92, 104, 122], with no clear distinction between the categories. Without conceptual clarity, such terminological variation blurs meaning and reduces analytical precision rather than improving readability.

A more insightful and clearer literature would ask what makes the barrier concept distinct, as well as what the barrier term does and means in both academic analyses and everyday contexts. Such moves could transform the vague notion of barriers into a more telling metaphor for the challenges of public involvement in health research.

### Barriers to what involvement?

Identifying barriers involves recognising and naming things that hinder involvement, but *understanding* them requires more. Many topical barrier analyses explicitly aim not only to identify barriers but also to understand them, and public involvement, better. It is therefore pertinent that topical barrier analyses become more curious about the involvement and engagement activities that are said to be obstructed by barriers.

In topical barrier analyses, barriers are said to obstruct everything from patient engagement to citizen science, co-design, and lived experience research. Unlike the barrier term itself, these forms of involvement are usually defined. One analysis, for example, defines patient engagement as the “active partnership between researchers, health care leaders, and health care service users (patients and/or patient partners) during the design, conduct, and dissemination of research” [76]; another defines user involvement as the “active partnership between the public and researchers in the research process, rather than the use of people as the ‘subjects’ of research” [62]; and a third defines co-design as a methodology which “actively engage specific groups of individuals, such as end-users, to aid in the development of products or services through knowledge sharing” [64]. While these definitions create an appearance of clarity, it remains an open question what it is exactly that barriers obstruct.

This is because topical barrier analyses do not connect with the recurring debates over which research activities “truly” and “meaningfully” engage the public and which are mislabelled. The broader literature repeatedly notes that participatory definitions are applied inconsistently and that terms are used interchangeably [134–139]. These debates partially persist for practical reasons – participatory relationships are organised and evolve in many ways [3, 140] – but they also have a normative aspect: different rationales for public involvement and engagement carry conflicting implications for what qualifies as involvement [4]. As a result, what is dismissed as tokenism may sometimes reflect clashes between different visions of involvement more than deliberate attempts to marginalise the public or engage them only in a box-ticking manner.

Currently, the definitions of public involvement that barrier analyses provide tell us what the hopes and ambitions are. Barrier analyses would generate more insights if they asked what precisely lies on the other side of each barrier, and at what point sufficient, or the right, barriers have been addressed for involvement to become meaningful rather than tokenistic or unfeasible.

### Involvement in what health research?

Another aspect that topical barrier analyses hardly engage with is the health research context in which public involvement takes place. Health research varies substantially across scientific disciplines, methodological approaches, and the physical settings in which it takes place. We should therefore expect these differences to pose distinct barriers (or variations thereof) and thereby create different spaces for public involvement. Nevertheless, topical barrier analyses treat health research as uniform.

Topical barrier analyses do address barriers across diverse health research contexts. They cover various health research fields such as rheumatology [133], paediatrics [105], palliative care [68, 100], drug development [83], and implementation science [99], and identify barriers across the translational pipeline, from preclinical laboratories [63, 127] to rural communities where health care is offered [129] and virtual spaces [114, 132]. Barriers have been found across research phases, from priority setting [85] to dissemination and implementation [65, 141, 142], and across countries and world regions, from Sub-Saharan Africa [73, 117], Europe [115], and Australia [74, 80] to the USA [86] and China [95]. Everywhere, barriers remain strikingly similar.

This similarity is unrealistic. For decades, science and technology studies has shown that the science*s* are characterised by distinct *epistemic cultures* [143], with different assumptions about which objects, topics, methods – and people – matter, and in what sense. Qualitative researchers, for example, openly discuss differences and similarities between their methods for qualitative inquiry and participatory research, such as what distinguishes doing a focus group study and engaging a public advisory group [144–146]. Ask an epidemiologist about the difference between their statistical methods and involving members of the public in their research; see if they can keep a straight face. It should be obvious that different researchers perceive, assess, and value public involvement and engagement differently, and we should expect the same goes for the barriers they experience.

Take the barriers concerned with the *representativeness* of patients and members of the public. They too illustrate that the scientific context matters little in topical barrier analyses. One analysis raises, for instance, concerns about patients’ “high degree of interest through personal experience” [31] and another warns that members of the public “may become experts over time” [70]. The issue often revolves around generalisability, sample sizes, objectivity, bias, partiality, and randomisation [31, 64, 70, 75, 82, 83, 117], which align more closely with positivist scientific paradigms than with interpretive or emancipatory paradigms concerned with diversity, social inclusion, and empowerment. From the latter perspectives, evidence may matter less – or matter differently – simply because involvement is the right thing to do [147], and people’s personal interest and experiences may be viewed more as a facilitator than a barrier to engagement. One topical barrier analysis recognises that there are varying interpretations of diversity and quotes an informant as saying that “… I am not sure I know what the diversity word seeks to address or cover” [132].

Future barrier analyses should look more closely at how barriers to involvement vary across research fields, settings and paradigms; how these barriers are constructed in some places more than in others; and what spaces for involvement they leave open.

### When and how do barriers develop?

Although a lack of time is one of the most frequently reported barriers to public involvement, topical barrier analyses treat time as a static factor rather than a dynamic condition. Thus, topical barrier analyses do not offer the timely insights we need to anticipate when specific barriers are likely to arise, how they may change, and how we may plan accordingly.

Occasionally, topical barrier analyses do acknowledge that barriers have a past, albeit a vague one. Some, for example, state that barriers to involvement relate to historical mistrust and exploitation [73, 75, 112, 113, 119], which are sometimes directly ascribed to the “negative legacy of unethical research” [86]. Here, the historical roots of barriers are invoked but not fully explained, and their present-day effects and meanings remain unspecified.

Other topical barrier analyses make attempts to account for the passage of time, usually within the timeline of research projects. One analysis distinguishes between barriers encountered “so far” in a specific partnership and barriers anticipated in a speculative future [88]. Another analysis maps barriers to user involvement across stages of involvement: from recruitment, through sustained involvement, to the conduct of research activities [62]. In this analysis, one barrier during the recruitment phase was that certain ethnic groups were reluctant to get involved because they felt ‘over-researched’, and a later barrier reported for the research phase was that researchers underestimated people’s desire for greater involvement [62]. As the title of one reviewed barrier analysis reads, we get only “snapshots” [80] of public involvement and engagement in topical barrier analyses.

A snapshot merely shows that a barrier was there, but barrier analyses would generate greater insight into public involvement if they showed us what change looks like and when and how it may come about.

### Barriers for whom?

When topical barrier analyses ask what the barriers are, they pay little attention to – and give little voice to – those who are affected by these barriers. The overall result is a picture more consensual than people’s experiences warrant, and less insightful than needed.

Even when focusing on the involvement of people with specific health conditions whose experiences might shape their engagement with research, differences fade. Some analyses simply conclude that the barriers they identified are “similar to barriers identified in other reviews or studies involving other patient groups” [62], “do not seem to be unique” [66], or “align closely with those identified in overall public and patient engagement” [101]. Occasionally, a few group-specific barriers are noticeable, but due to the topical format they are described only in passing. The reductive tendency is so strong that most analyses unproblematically present a single list of barriers almost as if barriers are the same for everyone involved.

A few topical barrier analyses do show that perspectives on barriers can differ within groups. Faithfull et al. are worth highlighting, as they found that the “more complex an understanding the researchers had of youth participation, the more barriers and challenges they could identify, and the less confident they felt engaging young people effectively. However, these participants were also more likely to consider youth participation an essential component of research” [54]. Two further analyses narrow in on barriers to patient partners in the publication process [126] and to their compensation [56] to illustrate and discuss challenges that disproportionately affect members of the public. While such elaborations move some group-specific nuances into the foreground, within-group differences are neglected as if people’s identities fitted a single category. There are experts and laypeople, and no one seems to fall out of this binary. Experts are not seen as individuals who might live with the conditions they study; members of the public are not seen as bringing academic skills from their education or professional experiences. So, while it is often said that one size does not fit all when it comes to public involvement, topical barrier analyses tend to both depict barriers as one-dimensional units from a single perspective and present people as stereotypes.

It would make barrier analyses more insightful if they moved beyond the expert–layperson binary and considered what other perspectives might be worth seeking out. Doing so could reveal greater depth and a wider range of viewpoints, some complementary and others potentially conflicting, thereby offering new angles on the challenges associated with public involvement.

### What are the functions of facilitators and recommendations?

The topical barrier analyses frequently declare an objective of giving recommendations for public involvement in health research, and, whether they do so or not, they tend to conclude with some advice for improvement. However, it is often unclear which facilitators and recommendations are supposed to address which barriers and, even more so, how.

One reason is the frequent mirror-image fallacy whereby barriers and facilitators reflect the same issue but one negatively and the other positively. Recall the topical barrier analyses that defined barriers as factors that disadvantage, challenge, prevent, or impede involvement. In contrast, the same analyses define *facilitators* as factors that advantage [77] or support [86, 128] or promote [128] involvement, making these factors conceptual opposites of barriers.

The conceptual flaw returns in the empirical analyses. One topical barrier analysis, for example, promotes the institutionalisation of research partnerships as a facilitator in parallel with a lack of institutionalisation presented as a barrier [86]. This mirror tendency is ingrained in the numerous barriers that are presented with the ritualistic phrase “a lack of X”: a lack of understanding or awareness [78, 80, 131]; of training [63, 75, 80]; of resources [59, 65, 80, 84], funding [80, 88, 106], and time [74]; of evidence and literature [63, 64, 74, 80]; of diversity [72, 74] and inclusion or inclusivity [69, 123]; of tools [85] and guidelines [87]; of continuity [57]; of a national strategy [75], sense of purpose [59]; confidence [62]; and of perceived appreciation [97] – to mention only some. The implication is straightforward: what is needed is more understanding, more training, more resources, more funding, more time, more evidence, more diversity, more tools, more continuity, more strategy, more purpose, and more confidence. The solution is the problem – just more of it.

The mirror also reveals what is absent. Many facilitators – and recommendations – do not address any specific barrier, and many barriers do not have clearly matching facilitators. This leaves it unclear whether, for some barriers, there is no way forward; whether facilitators are meant to balance and outweigh barriers as a whole, rather than match specific barriers; or whether specific configurations of barriers and facilitators make involvement more meaningful, while others are more likely to lead to tokenism.

It would be a modest but useful step forward for barrier analyses to consider and explain the relationships between barriers and facilitators, as well as the implications for the recommendations that follow.

### What is recommended?

Whatever the relationship between barriers, facilitators and recommendations, topical barrier analyses might still be useful if they provide actionable advice. For topical barrier analyses concerning public involvement, the problem is that facilitators and recommendations tend to be too abstract to guide action or too basic to do more than tinker with details.

Abstract recommendations offer no clear line of action. This is evident when topical barrier analyses recommend that communities should be mobilised “through targeted sensitization campaigns to ensure that community members understand the research objectives and deliverables” [73]; that “all co-investigators are protected within a relevant framework, with the potential to remain flexible during the co-design process in setting-specific situations” [103]; and “rigorous macro and micro level infrastructure with appropriate policy and governance mechanisms to support successful and meaningful patient partnership beyond a singular study” [57]. Such recommendations are of an epistemological nature that transcends concrete applicability by epitomising a position closer to academic ideation than actionable praxis.

Other recommendations suggest micro-actions. One topical barrier analysis, for example, recommends that reading materials for members of the public be written in large, black font on coloured paper [70] and another recommends that involvement can be sustained through thank-you notes, Christmas cards, and telephone calls [62]. Yet another recommends that researchers “[a]sk the right questions and be ready to listen” because “[i]f you ask the right questions, patients will answer them” [83]. As simple as that.

To be fair, most recommendations appear more reliable: be flexible, secure adequate funding and time, and ensure appropriate training and support for researchers and co-researchers, for example. But flexible to what extent? How much funding is adequate, and how much time is sufficient? What skills and support are relevant? Topical recommendations are aspirational at best and platitudes at worst.

Recommendations could become more actionable if they specified who is to act, what is to be done, and why it would lead to a meaningful improvement. Authors who find that other ways are better for formulating advice could strengthen the literature by explaining their choice and reasoning.

### What can members of the public do?

Public involvement is about expanding and diversifying the roles that members of the public take on in the health research that may ultimately affect their lives. Topical barrier analyses, however, are far less interested in what laypeople can do to access and shape health research on their own terms than in how their participation can be managed by professional researchers.

Consider again the recommendations discussed above. Who should mobilise communities through targeted sensitisation campaigns and ensure that community members understand the research objectives and deliverables? Who should protect co-investigators within a relevant framework while remaining flexible? Who should incorporate policy and governance mechanisms into their macro- and micro-level infrastructures to support patient partnerships? Who should provide reading materials in large black font on coloured paper and sustain involvement through thank-you notes, writing Christmas cards, and making telephone calls? Who should ask the right questions and listen because *patients* will answer them?

Topical barrier analyses expect researchers, project leaders, and their institutions to act. Members of the public must look elsewhere for advice. This asymmetry is easily missed because the analyses are written in an academic style that relies on the passive voice, leaving the acting subjects unspecified. When reading with attention to the implied actors, the recommendations appear attuned to helping researchers anticipate and reduce friction so research projects can proceed smoothly. While this is a legitimate goal, the analyses ultimately neglect the people who seek to influence health research.

At first, this reading may seem somewhat unfair. After all, the present review shows that members of the public co-authored almost half of the topical barrier analyses reviewed, and in that sense these studies meet the ideal of being conducted with or by the public rather than to, about or for them. However, what it also reveals is that co-authorship does not in itself ensure that the results and recommendations are oriented towards the needs or interests of members of the public.

One topical analysis does more by including a brief paragraph with recommendations explicitly addressed to members of the public [77]. Among these recommendations is the advice that members of the public “should be aware that responding to written consultations may not be a good use of their time”. There is not a lot they can, or should, do, it seems.

A pressing challenge for future barrier analyses in the domain of public involvement is therefore to produce something that speaks not only to those already occupying positions of authority, but also to those attempting to move from the margins to the centre of health research.

## Discussion

There is a high risk that future topical barrier analyses will reinforce what many people already know: that there are many reasons why public involvement in health research can be challenging. The repetitive and commonsensical state of the topical barrier literature is the consequence of an analytical approach that does little more than present easily recognisable factors as being either good or bad for public involvement. It is caused by an obsession with identifying the *range* of issues that impede involvement alongside good intentions towards advancing public involvement in health research.

The potential for future analyses to become more insightful and useful is equally great. To this end, barrier analyses must be redirected towards new inquiries. Table 2 proposes eight lines of inquiry with potential sub-questions that may move aspiring authors beyond the current analytical limitations of topical analyses of barriers and facilitators to public involvement. Each line corresponds to the critical appraisal presented in the previous section, with five focusing on questions that may make barrier analyses more insightful and three on questions that may make them more useful. Together, the first five inquiries should move us beyond barriers as readily described by informants towards something that is not immediately apparent in, yet strongly resonates with, their experiences. The last three lines of inquiry call for something more than generally good advice: evidence-informed recommendations that expand people’s agency, and, most urgently, members of the public’s agency.

**Table 2:** Lines of inquiry to move barrier analyses beyond their current shortcomings

**What is a barrier?** • How can barrier (and facilitator) concepts be better defined and operationalised? • What does the term “barrier” mean to people who take part in public involvement, and how do they use the term in everyday contexts? • What would we learn from developing different barrier metaphors (e.g. road bumps, border controls, or labyrinths) to prompt and tell richer stories about involvement?
**Barriers to what involvement?** • What exactly is on the other side of each barrier to public involvement? • At what point are enough, or the right, barriers addressed for public involvement to become meaningful? • Which, or how many, barriers cause involvement to become tokenistic, unfeasible, or unwarranted?
**Involvement in what health research contexts?** • How do various research fields spur similar and distinctive barriers to public involvement? • How do different scientific settings (e.g., laboratories, clinics and communities) pose similar and distinctive barriers, and what space remains for involvement? • How do various research paradigms, traditions, and practices lead people to encounter – or create – some barriers more than others?
**When and how do barriers develop?** • What is the process by which a barrier to public involvement develops and changes, or not? • When do barriers arise, change, persist, and fall? • To what degree can barriers and their development be expected or predicted, and therefore mitigated, in advance?
**Barriers for whom?** • How are people affected differently by barriers to public involvement? • Whose perspectives, other than those of research experts and laypeople, would be relevant to examine? • What differences and disagreements are there regarding barriers between, and especially within, social groups?
**What are the functions of facilitators and recommendations?** • Which facilitators and recommendations address which barriers to public involvement? • Which barriers remain unaddressed, and why? • Are facilitators and recommendations meant to address specific barriers, to balance barriers as a whole, or to create configurations that shape whether involvement becomes meaningful or tokenistic?
**What is recommended?** • Who should act, what exactly should be done, and why should it work? • What is the appropriate amount or strength of suggested countermeasures against identified barriers to public involvement? • What makes a recommendation useful?
**What can members of the public do?** • What can members of the public, individually and collectively, do to overcome barriers to involvement on their own terms? • When should they challenge barriers to involvement, and when should they work within the space that exists? • When might the best advice for members of the public be to disengage from, or not engage in, health research projects?

The questions in Table 2 are not a definitive agenda for future research, nor should they be treated as an exhaustive checklist that every barrier analysis must attend to. On the contrary, in-depth engagement with one question or a small number of questions is likely to expand our knowledge and generate something more useful than a superficial run-through of several questions.

Beyond public involvement, scholars who have evaluated barrier analyses already offer some responses to the questions this review presents. They have, for example, proposed elaborate definitions of the concept of barrier [37, 42]. Mostly, however, these scholars have focused on advancing the conceptual and methodological basis of barrier analyses. Although this review has not undertaken a full appraisal of the concepts and methods used in topical barrier analyses, conceptual clarity and methodological rigour are valuable – also in the context of public involvement. But on their own, if analyses of barriers to public involvement continue to ask the same narrow questions, conceptual and methodological advances will only change how findings that remain all too familiar are generated.

Still, diversifying the questions asked in barrier analyses may have methodological implications. Analyses oriented towards explaining, predicting, or intervening to address – rather than enumerating – barriers to involvement will require approaches that are less straightforward and less topical. This is where the tension in public involvement arises: more complex methodologies may have higher academic merit, but they may also exclude some members of the public from the process or alienate them from the results. It is therefore worth discussing when topical barrier analyses retain some relevance, despite their shortcomings.

As observed in this review, most topical barrier analyses seem to be the only ones within their respective journals, which shows that the approach resonates widely. This could also indicate that barrier analyses are used to raise awareness about public involvement in research areas where participatory research approaches are uncommon or unconventional. In such contexts, topical barrier analyses may be a way to acknowledge public involvement and its challenges without allowing these challenges to become an excuse for maintaining a minimal role in health research for members of the public.

By contrast, the credibility of topical barrier analyses is more questionable in journals specifically dedicated to public involvement and engagement. Here too, topical barrier analyses continue to accumulate without analytical progress. On the one hand, journals promoting public involvement may function as important outlets for studies rejected by journals less receptive to research with public involvement. On the other hand, journals committed to public involvement arguably bear a special responsibility not only to advance involvement but also to treat it as a research object in its own right. This involves pushing for methodological innovations and actively encouraging, if not requiring, that research builds on, complements or corrects existing knowledge – rather than merely rephrasing and restating it over and over.

Finally, there may be other benefits of undertaking topical barrier analyses, some of which may escape the published reports. One possibility is that the benefits are less about the published paper and more about the analytical process. Perhaps identifying and discussing barriers and facilitators provides authors, including members of the public and researchers, with an opportunity to collaborate on something relatively simple. In doing so, they may develop trust, skills, and shared understanding – factors that have been highlighted as facilitators of involvement – which help them engage in more demanding projects together. In a similar way, the process might, in some cases, make people realise that further collaboration is undesirable, allowing them to direct their efforts elsewhere. Thus, as a process, analysing barriers and facilitators in a topical manner may still hold relevance. Even so, the questions proposed by this review may enhance the value that members of the public and researchers gain from, and offer to others through, undertaking yet another analysis of barriers to and facilitators of public involvement in health research.

### What alternatives may look like

Focusing on the topical type of barrier analysis that dominates the literature required me to set aside a couple of exceptional barrier analyses. Although excluded from the formal review, they are worth highlighting because they illustrate some of the directions future barrier analyses could take to become more insightful and useful.

The first is a study by Northway, Howarth and Evans, which describes how ethical approval was an initial barrier for their participatory research project involving people with intellectual disabilities [148]. In their paper, the authors describe, step-by-step, how they engaged both the ethics committee and their funders to jointly identify an acceptable route forward that upheld both ethical and scientific standards while enabling maximum engagement by co-researchers. In this account, the barrier is not depicted as a monolithic obstacle but as a negotiable social contract. The study presents a coherent public involvement process rather than a topical fragmentation of it. In doing so, Northway, Howarth and Evans approach several questions that this review encourages. These include what a barrier is and how it functions; when it is encountered, who placed it there and for what *legitimate* reasons; and which actions and considerations may help to overcome – or, perhaps more tellingly, bend – the barrier encountered.

Another notable barrier analysis is authored by Hawke et al. and evaluates the impact of a workshop to increase researchers’ capacity to engage young people in health research [149]. This study compares how frequently researchers reported eleven named barriers before and after they attended the workshop. The statistical analysis shows that four barriers decreased significantly, six were unaffected, and one – namely, “Don’t have time or human resources to support this [youth engagement]” – increased significantly following the intervention. In doing so, Hawke et al. approach questions around when barriers are present, when they diminish, and which actions influence them to what degree. The study also gives an intriguing lesson: that well-designed training programmes in public involvement – which are often recommended by topical barrier analyses – may have both intended and unintended consequences.

For some readers, the study by Northway, Howarth and Evans likely illustrates a barrier analysis that is more insightful than topical ones, and, just as the study by Hawke et al., likely illustrates an analysis that is more useful. However, the distinction between insightfulness and usefulness should not be applied too strictly. A processual account such as Northway, Howarth and Evans’s is useful too: it describes barriers as we experience them most often on a developing timeline in which our actions have some effect on and response from our surroundings. An impact evaluation like Hawke et al.‘s is insightful too: it reveals something about the world that we may not otherwise have noticed. Thus, future barrier analyses may emphasise one of the two aims over the other but will benefit from giving some thought to both the insightfulness and usefulness of their findings.

### Strengths and limitations

The main contribution that this review makes is that it breaks with the repetitive pattern it critiques. It does not ask yet again what the barriers are to public involvement in health research and how they may be overcome. Furthermore, it adds an important qualification to its criticism by specifying the kind of barrier analysis with which it is concerned: the topical. Other scholars who have critically appraised barrier analyses have occasionally noted their methodological diversity [36, 37], yet have proceeded to critique the literature as largely uniform – as if there were no important differences between quantitative, qualitative or mixed-methods research, or between positivist and interpretive approaches. This review suggests that most barrier analyses, at least those concerning public involvement in health research, adopt a topical approach that is broadly aligned with positivist research paradigms, albeit in a simplified form.

The initial search strategy drew on systematic search techniques applied across the foremost health research databases, but it also has limitations. One limitation is its reliance on relatively simple search strings, which seem to have been more effective at identifying barrier analyses with relatively generic public involvement terminology (i.e. the involvement and/or engagement of a social group) and less effective at identifying analyses using more unique participatory terms (e.g. action research, participatory research, or co-production). It is possible that a more comprehensive search strategy would have identified additional relevant papers, but it does not follow that the inclusion of such studies would have substantially altered the review’s findings or its propositions about how to move beyond the current shortcomings of topical barrier analyses. Indeed, the reviewed analyses that address barriers to, for example, citizen science [94], co-design [64], or lived experience research [60] do not stand out from the rest of the reviewed literature.

The decision to confine the literature search to the foremost health databases may be more significant. Other databases covering the social sciences and humanities (e.g. Web of Science, Sociological Abstracts, and Anthropology Plus) might contain barrier analyses that ask different questions and give answers that break with the tendencies in topical barrier analyses, and, if so, their inclusion in the review could have implications for the arguments I have developed. Nilsen’s recent appraisal of barrier analyses [38], for example, could indicate that analyses in implementation science may be drawing more consistently on established frameworks than is the case for analyses concerning public involvement. Thus, this review’s arguments clearly apply to barrier analyses regarding public involvement published in mainstream health research journals, but it is my impression they have relevance in other domains too.

Reviewing the literature also limits this paper to examining barrier analyses as they are reported in published studies. As discussed above, authors of barrier analyses may gain insights or be inspired to act during their work in ways that are not captured by the published reports. Future research could therefore examine what authors of barrier analyses do during and after their analytical process, with a particular focus on their actions and reasoning around public involvement and engagement in health research. Such research could point to additional questions and insights that have so far escaped the barrier literature but may be of value to those promoting active roles for members of the public in health research.

## Conclusion

Barrier analyses may become more insightful and useful for those who promote public involvement in health research if they start asking new questions that move beyond listing barriers and facilitators and giving vague advice for improvement. What is needed is, in most situations, not another topical barrier analysis that identifies familiar barriers and facilitators to public involvement because such analyses are already piling up. Adding another will only confirm, once again, that there are all sorts of reasons why public involvement and engagement can be challenging. We have known that for a long time – have we not?

What is needed is a more curious attitude towards how barriers can be viewed, understood, and navigated in different ways and in various situations. For researchers undertaking a barrier analysis, this means resisting the temptation to ask, once more, what the barriers are and how they may be overcome, and instead choosing one or two of the lines of inquiry proposed here – or others like them – and pursuing them in depth. For peer reviewers and editors, particularly at journals dedicated to public involvement and engagement, it means asking whether a submitted barrier analysis builds on, complements or corrects what is already known, rather than merely restating or reorganising it. For members of the public who are involved in making barrier analyses, it may mean insisting that the analysis speaks to their needs, especially through the recommendations that currently give members of the public very little to act on.

The additional challenge is to make barrier analyses more insightful without increasing their conceptual and methodological complexity to the point where they end up inaccessible to those without advanced social research expertise. This challenge and the significant shortcomings of barrier analyses should be taken seriously. If barrier analyses do not move beyond their repetitive tendency, then calls to abandon barrier analyses will become increasingly difficult to resist.

## Supplementary Information

Below is the link to the electronic supplementary material.


Supplementary Material 1


## Data Availability

No datasets were generated or analysed during the current study.
