# Supplementary material for: Questions for more insightful and useful analyses of barriers and facilitators to public involvement and engagement in health research: a critical narrative review
Source: Res Involv Engagem. 2026 Sep 1;12:137. doi: 10.1186/s40900-026-00963-9 (PMC13536805; doi:10.1186/s40900-026-00963-9)
Supplement: Supplementary file 1 [file 40900_2026_963_MOESM1_ESM.docx]

**Supplementary material for *Questions for more insightful and useful analyses of barriers and facilitators to public involvement and engagement in health research: A critical narrative review***

**Corresponding author**

Lauge Neimann Skov

**Author information**

Steno Diabetes Center Copenhagen, The Capital Region of Denmark, Borgmester Ib Juuls Vej 83, 2730 Herlev, Denmark

University of Copenhagen, Department of Public Health, Øster Farimagsgade 5, 1353 København K, Denmark.

Email: [lauge.neimann.skov@regionh.dk](mailto:lauge.neimann.skov@regionh.dk)

**The search string used for identifying relevant literature**

A search was developed based on the Population-Phenomenon-Context [PCC] mnemonic normally used for scoping reviews^1^:

- **Population:** The public broadly defined
- **Phenomenon:** Barriers
- **Context:** Involvement and engagement in health research.

# Information sources and search methods

1,839 records were found by searching the MEDLINE (via Ovid), Embase (via Ovid), CINAHL (via EBSCO), and PsycInfo (via EBSCO) on 14 June 2023. When removing duplicates, 1,402 records remained and were screened before the final sample of topical barrier analyses regarding public involvement and engagement in health research was assembled. (see flow diagram below). Additional records were identified and screened following an updated search on 29 to 30 June 2026.

## Database searches

The first search string was developed in MEDLINE (Ovid) and subsequently translated for the other databases.

All search strings and their results are reported in full below.

| **MEDLINE via Ovid** | | | |
| --- | --- | --- | --- |
| **#** | **Concept** | **Search string** | **Number of records** |
| 1 | **Population:**  The public | (adolescent$ or caregiver$ or care-giver$ or carer$ or child$ or citizen$ or client$ or communit$ or consumer$ or famil$ or parent$ or patient$ or peer$ or people$ or public$ or relative$ or resident$ or user$ or youth$).ti,ab,kw,kf. | 12,884,128 |
| 2 | **Context A:**  Involvement and engagement | (involv$ or engag$).ti,ab,kw,kf. | 2,893,883 |
| 3 | **Context B:**  In health research | (research$ or science$ or evidence$ or stud$ or rct$ or trial$ or review$).ti,ab,kw,kf. | 16,556,358 |
| 4 | **Phenomenon:**  Barriers | (barrier$).ti,ab,kw,kf. | 402,209 |
| 5 | **All combined:**  Barriers to public involvement and engagement in health research | (1 adj2 2 adj2 3) and 4 | 784 |
| 6 | **Updated search** | (1 adj2 2 adj2 3) and 4 | 1,639 |
| 7 | **Time range limit** | Limit 5 to (da="20230401-20261231" or dt="20230401-20261231" or ez="20230401-20261231" or ed="20230401-20261231") | 908 |
| 8 | **In total** |  | 1,692 |

| **Embase via Ovid** | | | |
| --- | --- | --- | --- |
| **#** | **Concept** | **Search string** | **Number of records** |
| 1 | **Population:**  The public | (adolescent$ or caregiver$ or care-giver$ or carer$ or child$ or citizen$ or client$ or communit$ or consumer$ or famil$ or parent$ or patient$ or peer$ or people$ or public$ or relative$ or resident$ or user$ or youth$).ti,ab,kw,kf. | 17,450,084 |
| 2 | **Context A:**  Involvement and engagement | (involv$ or engag$).ti,ab,kw,kf. | 3,719,988 |
| 3 | **Context B:**  In health research | (research$ or science$ or evidence$ or stud$ or rct$ or trial$ or review$).ti,ab,kw,kf. | 21,299,462 |
| 4 | **Phenomenon:**  Barriers | (barrier$).ti,ab,kw,kf. | 510,461 |
| 5 | **All combined:**  Barriers to public involvement and engagement in health research | (1 adj2 2 adj2 3) and 4 | 426 |
| 6 | **Updated search** | (1 adj2 2 adj2 3) and 4 | 1,016 |
| 7 | **Time range limit** | limit 6 to (dc="20260401-20261231" or dd="20260401-20261231" or rd="20260401-20261231") | 96 |
| 8 | **In total** |  | 522 |

| **CINAHL via EBSCO** | | | |
| --- | --- | --- | --- |
| **#** | **Concept** | **Search string** | **Number of records** |
| S1 | **Population:**  The public | TI (adolescent* OR caregiver* OR care-giver* OR carer* OR child* OR citizen* OR client* OR communit* OR consumer* OR famil* OR parent* OR patient* OR peer* OR people* OR public* OR relative* OR resident* OR user* OR youth*) OR AB (adolescent* OR caregiver* OR care-giver* OR carer* OR child* OR citizen* OR client* OR communit* OR consumer* OR famil* OR parent* OR patient* OR peer* OR people* OR public* OR relative* OR resident* OR user* OR youth*) | 3,594,083 |
| S2 | **Context A:**  Involvement and engagement | TI (involv* OR engag*) OR AB (involv* OR engag*) | 490,628 |
| S3 | **Context B:**  In health research | TI (research* OR science* OR evidence* OR stud* OR rct* OR trial* OR review*) OR AB (research* OR science* OR evidence* OR stud* OR rct* OR trial* OR review*) | 3,605,462 |
| S4 | **Phenomenon:**  Barriers | TI (barrier*) OR AB (barrier*) | 116,303 |
| S5 | **All combined:**  Barriers to public involvement and engagement in health research | (S1 N1 S2 N1 S3) AND S4 | 232 |
| S6 | **Updated search** | (S1 N1 S2 N1 S3) AND S4 | 654 |
| S7 | **Time range limit** | Limit to S6 to 04/2023 – 12/2026 | 251 |
| 8 | **In total** |  | 483 |

| **PsycInfo via EBSCO** | | | |
| --- | --- | --- | --- |
| **#** | **Concept** | **Search string** | **Number of records** |
| S1 | **Population:**  The public | TI (adolescent* OR caregiver* OR care-giver* OR carer* OR child* OR citizen* OR client* OR communit* OR consumer* OR famil* OR parent* OR patient* OR peer* OR people* OR public* OR relative* OR resident* OR user* OR youth*) OR AB (adolescent* OR caregiver* OR care-giver* OR carer* OR child* OR citizen* OR client* OR communit* OR consumer* OR famil* OR parent* OR patient* OR peer* OR people* OR public* OR relative* OR resident* OR user* OR youth*) | 2,735,146 |
| S2 | **Context A:**  Involvement and engagement | TI (involv* OR engag*) OR AB (involv* OR engag*) | 735,656 |
| S3 | **Context B:**  In health research | TI (research* OR science* OR evidence* OR stud* OR rct* OR trial* OR review*) OR AB (research* OR science* OR evidence* OR stud* OR rct* OR trial* OR review*) | 3,658,324 |
| S4 | **Phenomenon:**  Barriers | TI (barrier*) OR AB (barrier*) | 91,095 |
| S5 | **All combined:**  Barriers to public involvement and engagement in health research | (S1 N1 S2 N1 S3) AND S4 | 397 |
| S7 | **Updated search** | (S1 N1 S2 N1 S3) AND S4 | 640 |
| S8 | **Time range limit** | Limit to S6 to 04/2023 – 12/2026 | 217 |
| 8 | **In total** |  | 614 |

^1^ Peters, M. et al. Updated methodological guidance for the conduct of scoping reviews. JBI Evidence Synthesis 18(10):p 2119-2126, October 2020. DOI: 10.11124/JBIES-20-00167.
